# Mutational spectrum of DNA damage and mismatch repair genes in prostate cancer

**DOI:** 10.3389/fgene.2023.1231536

**Published:** 2023-09-04

**Authors:** Fidelis Charles Bugoye, Rispah Torrorey-Sawe, Richard Biegon, Nazima Dharsee, Fidelice M. S. Mafumiko, Kirtika Patel, Simeon K. Mining

**Affiliations:** ^1^ Government Chemist Laboratory Authority, Directorate of Forensic Science and DNA Services, Dar es Salaam, Tanzania; ^2^ Department of Pathology, Moi Teaching and Referral Hospital, Moi University, Eldoret, Kenya; ^3^ Ocean Road Cancer Institute, Dar es Salaam, Tanzania

**Keywords:** mutation, DNA damage, prostate cancer, mismatch, repair-deficient, landscape

## Abstract

Over the past few years, a number of studies have revealed that a significant number of men with prostate cancer had genetic defects in the DNA damage repair gene response and mismatch repair genes. Certain of these modifications, notably gene alterations known as homologous recombination (HRR) genes; *PALB2*, *CHEK2 BRCA1*, *BRCA2*, *ATM*, and genes for DNA mismatch repair (MMR); *MLH1*, *MSH2*, *MSH6*, and *PMS2* are connected to a higher risk of prostate cancer and more severe types of the disease. The DNA damage repair (DDR) is essential for constructing and diversifying the antigen receptor genes required for T and B cell development. But this DDR imbalance results in stress on DNA replication and transcription, accumulation of mutations, and even cell death, which compromises tissue homeostasis. Due to these impacts of DDR anomalies, tumor immunity may be impacted, which may encourage the growth of tumors, the release of inflammatory cytokines, and aberrant immune reactions. In a similar vein, people who have altered MMR gene may benefit greatly from immunotherapy. Therefore, for these treatments, mutational genetic testing is indicated. Mismatch repair gene (MMR) defects are also more prevalent than previously thought, especially in patients with metastatic disease, high Gleason scores, and diverse histologies. This review summarizes the current information on the mutation spectrum and clinical significance of DDR mechanisms, such as HRR and MMR abnormalities in prostate cancer, and explains how patient management is evolving as a result of this understanding.

## 1 Introduction

Prostate cancer (PCa) is the most common cancer among men worldwide and a significant public health burden. The disease is diagnosed more frequently than any other type of cancer (Sanjose et al., 2011; Lozano et al., 2012; Sung et al., 2021). Previous studies (Siegel et al., 2011; El Noor and El Noor, 2015; Bugoye et al., 2019), have shown that PCa is the most frequent solid tumor in males and one of the main causes of cancer-related deaths in men worldwide. The incidence and mortality of PCa are alarming, 2,293,818 new cases are anticipated to be diagnosed between now and 2040, and a 1.05% rise in mortality is expected (Rawla, 2019). Incidence and death of PCa are both associated with age at the time of diagnosis. African-American men have higher incidence rates than white men. Previous researchers reported 158.3 new cases per 100,000 African-American men and twice the mortality rate compared to reported data in white men, and the disparity is caused by a variety of social, environmental, and genetic factors (Panigrahi et al., 2019). PCa progression is typically gradual and symptom-free, and treatment may not even be necessary, but most frequent complaints are difficulty urinating, increased urination frequency, and nocturia, though prostatic enlargement may induce similar symptoms. The advanced stages of the disease may appear with back discomfort and urinary incontinence because the axial skeleton is the most usual site of bone metastatic sickness.

In 2018, 1,276,106 new cases of PCa were reported worldwide, making up about 7.1% of all male malignancies (Bray et al., 2018; Rawla, 2019). Prostate cancer incidence rates vary by geographic area, and these variations have been connected to a range of modifiable and non-modifiable factors (Rawla, 2019; Sung et al., 2021). Smoking, food, obesity, and exposure to chemicals are all aspects of lifestyle that can be modified to lower the chance of developing prostate cancer. These risk factors can be changed or managed to reduce a person’s chance of developing the illness. Age, race/ethnicity, family history, and some genetic alterations such as mutations in the Homologous recombination-related DNA Damage Repair genes *PALB2*, *CHEK2*, *BRCA1*, *BRCA2*, *ATM*, and *MLH1*, *MSH2*, *MSH6*, *PMS2* Mismatch Repair (MMR) genes are risk factors that cannot be modified to reduce risk of developing disease (Lang et al., 2019; Sedhom and Antonarakis, 2019; van Wilpe et al., 2021). In this review, the Mutation spectrum of HRR genes, and DNA MMR genes involved in DNA damage repair mechanisms and their therapeutic applications are discussed.

## 2 Materials and methods

The peer-reviewed articles in this review spanned the time between January 2000 and June 2023 and were taken from PubMed and Google Scholar. The search terms “prostate cancer OR genetic alteration OR mutation variant OR gene OR DNA Damage repair OR individual OR Mismatch repair OR Homologous recombination OR immunotherapy OR prevalence OR frequency OR Genetic OR Spectrum OR Landscape” were used. 1,222 publications from Google Scholar and 12,773,760 articles from Pubmed made up 12,774,982 total results for our search phrase. Since broad key terms were employed, the search string’s filtering mechanism also supplied irrelevant results. The following inclusion criteria were used to manually screen retrieved articles: 1) Articles describing a phenotypic or genetic condition associated with prostate cancer 2) Articles that detail genetic variations 3) Articles describing gene changes related to DNA damage repair 4) Articles outlining genes involved in homologous recombination repair (HRR) 5) Articles discussing DNA damage repair modifications and the clinical or therapeutic use of the Mismatch Repair (MMR) gene. To retrieve and validate the reported Mutations, Either ClinVar, European Nucleotide Archive (ENA), DNA Databank of Japan (DDBJ), or Gene Bank Genetic Variants databases were used. Also, with the exception of two articles published in 1999 and 1998, all other selected articles published in 2000 and above were considered. However, articles with irrelevant information for this review and those with redundant information and duplicate information were not selected. Thus, in this review, 174 articles were used.

## 3 Race and ethnicity in prostate cancer

Hereditary factors are known to play a role in the higher mortality and incidence rates of PCa in African American males compared to European American men (DeSantis et al., 2016). Greater genomic mutation, which results in a more aggressive phenotype, is one of the characteristics of the ethnic disparities in PCa biology. Knowing how to target genetic anomalies like DDR gene mutations offers the way to efficient treatments that can improve clinical outcomes (Kohaar et al., 2022; White et al., 2022). Compared to white males, men with African heritage and men from South America continue to have higher incidence and mortality rates of PCa (Belkahla et al., 2022). Men from the Middle East, North Africa, and Asia often have the lowest incidence of PCa in comparison to other ethnic groups, such as non-Hispanic White males, who have a chance of PCa diagnosis of 1 in 8 men over their lifetime, data from the National Cancer Institute suggest that men of African descent often have the greatest rate (1 in 6 men) (Akaza et al., 2011; Powell and Bollig-Fischer, 2013). A number of factors, including the downregulation of DNA repair genes in African men, contribute to the increased incidence and mortality rate of prostate cancer in African men. When compared with Non-Hispanic white, African American men were found with upregulation genes governing inflammatory pathways including *CCL4*, *IFNG*, *CD3*, *IL33*, and *ICOSLG* despite the downregulation of DNA damage response mechanisms (DeSantis et al., 2016; Nair et al., 2022). However, it should be highlighted that race is a social rather than a biological construct; in PCa, there have been conflicting results and challenges to demonstrate the prevalence and differences in DNA repair gene mutation between races, and results ought to be handled with caution (Reizine et al., 2023).

## 4 Challenges and controversies in the management of prostate cancer

The biological heterogeneity of PCa is the root of many of the difficulties in treating the condition. Due to the clinical tumor presentation, genetic or phenotypic variability, and therapy outcomes, PCa has been described as a heterogeneous illness (Tolkach and Kristiansen, 2018; Haffner et al., 2021). Because of these heterogeneities, there is no one-size-fits-all approach to treating PCa. This has led to controversies over how to treat patients (Shore et al., 2022). For instance, the issue of whether routine PCa screening is more advantageous than the risk of over-detection and over-treatment is still up for debate and necessitates individualized treatment approaches (Force, 2018; Shore et al., 2022). Despite the promise of precision therapy in the treatment of PCa has increased due to advances in genetic testing, the use of genetic data by practitioners in developing countries is hampered by issues with insufficient capacity, the number of patients requiring genetic testing, and accessibility (Szymaniak et al., 2020). Similar to this, worries about overtreatment have changed recommendations for active monitoring, and physicians’ approaches to managing PCa vary widely (Shore et al., 2022). According to research, more than 50% of respondents disagree with management concerns relating to PCa which is advancing (Saad et al., 2019). These findings, along with others of a similar nature, have highlighted the necessity of improved knowledge of the genetic mutation spectrum in prostate cancer (Finch et al., 2022) and other concerted efforts to improve the illness management (Gillessen et al., 2015; Gillessen et al., 2018; Saad et al., 2019; Gillessen et al., 2020). Genetic Mutation in Prostate Cancer.

Genetic and epigenetic changes normally occur at different levels, and these genetic modifications have provided potential applications as biomarkers in cancers. The genetic origins of prostate cancer have been the subject of research for decades. This is because genes involved in the DNA damage repair pathways can increase the risk of developing PCa (Robinson et al., 2015a). In Cells, there are sophisticated networks of non-redundant mechanisms, including base alterations, strand breaks, and interstrand crosslinks, these mechanisms detect and repair DNA damage. Direct repair, base excision repair, nucleotide excision repair, mismatch repair (MMR), homologous recombination repair (HRR), non-homologous end joining (NHEJ), and the Fanconi anemia repair pathways are important DNA damage repair mechanisms. According to research, this PCa is substantially more likely to be developed in carriers of HRR and MMR gene mutations (Robinson et al., 2015a).

### 4.1 Mismatch repair gene mutations

A post-replication technique called DNA mismatch repair (MMR) is used to correct base mismatches and replication-related insertion or deletion mistakes. Genetic instability is brought on by a loss of MMR gene function resulting in a buildup of errors that typically occur during DNA replication (Chen et al., 2001). The risk of PCa in men with MMR gene mutations has been demonstrated to be significantly enhanced (Rantapero et al., 2020). Individuals that have MMR mutations are typically predicted to account for 2%–5% of PCa cases (Ritch et al., 2020; Ye et al., 2020), and they are generally identified by their Gleason score 8 and *de novo* metastases (Dominguez-Valentin et al., 2016; Ye et al., 2020; Jiang et al., 2021; Graham and Schweizer, 2022). The genetic mutations in MMR genes in the prostate may aid in understanding PCa carcinogenesis, which may have additional repercussions for ICBs and other types of treatment. This review effort focuses on the *MSH2*, *MSH6*, *MLH1*, and *PMS2* genes (Table 1), which are more frequently found in PCa and have recently been described by Pecina et al., though previous authors have reported on other MMR genes which are also connected to high microsatellite instability and high tumor mutational load involved in PCa progression (Chen et al., 2011; Sedhom and Antonarakis, 2019; Pećina-Šlaus et al., 2020).

**TABLE 1:** Summary of mismatch repair genes related to PCa.

Gene	Chromosome	Total number of Exon coverage	Prevalence of Germline Mutation in mCRPC	Reported Mutation	References
*MLH*1	3	19	0–0.9%	Del exon 19 c.350C>T	Fukuhara et al. (2014); Haraldsdottir et al. (2014); Dominguez-Valentin et al. (2016); Wilczak et al. (2017); Sharma et al. (2020); Javeed et al. (2022)
				c.588+5G>A	
				c.1537_1547delinsC	
				c.1667+2delTAAATCAinsATTT	
				c.1732-2A>T	
				c.1852_54delAAG	
				c.1310del	
*MSH*2	2	16	1–2%	c.942+3A>T	Haraldsdottir et al. (2014); Pritchard et al. (2014); Pritchard et al. (2016); Le et al. (2015); Dominguez-Valentin et al. (2016); Abida et al. (2017); Ye et al. (2020); Chang et al. (2022)
				Del exons 1-6	
				Del exon 8 c.2038>T	
				c.1906G>C	
				c.560G>T	
				C.892C>T c.1786_788del AAT	
				c.2347del C	
				C2orf61343kb inversion	
				FBXO11 inversion	
				KCNK12 74kb inversion	
				KCNK12 40kb inversion	
				Exons 8-16 del c.1082del	
				c.1124_1125insG	
				c.2364_2365insTACA	
*MSH*6	2	10	1%	c.3155_315delAG	Haraldsdottir et al. (2014); Pritchard et al. (2014); Pritchard et al. (2016); Mateo et al. (2015); Wilczak et al. (2017); Ye et al. (2020); Chang et al. (2022); Cheng et al. (2023)
				c.3261dupC	
				c.1444C>T	
				c.1483C>T	
				c.3647-1G>A	
				c.3609_3612delTGCA	
				c.3992+1T>C	
				del exon 8 to 3′ UTR	
				c.3799_3800del	
				c.3573del	
*PMS*2	7	15	0.29–0.4%	Del exons 11-12	Haraldsdottir et al. (2014); Pritchard et al. (2016); Wilczak et al. (2017); Chang et al. (2022); Cheng et al. (2023)

#### 4.1.1 MutL homolog 1

The MutL homolog 1 (*MLH*1) protein, a member of the MutL family of DNA mismatch repair proteins. *MLH1* consists of 56kilobases is located in chromosome 3 and consists of 11 exons for coding *MLH*1 Protein which recognizes incorrect nucleotides, causing them to be expelled and replaced in a strand-directed manner (Fukuhara et al., 2014; Zhen et al., 2018). Several PCa cohorts have been examined for the breadth of *MLH1* gene mutations using sequencing data (Pande et al., 2012; Haraldsdottir et al., 2014; Dominguez-Valentin et al., 2016). *MLH*1 mutations were found in 0.7% of the 150 mCRPC patient (Robinson et al., 2015b). Additionally, PCa with the *MLH1* gene mutation has been connected to an elevated acute form of the illness, a higher Gleason score, a lack of differentiation, and a higher rate of distant metastasis (Shenderov et al., 2019; Antonarakis et al., 2020). Frameshift, deletion, and missense mutations are additionally the most prevalent *MLH*1 mutation types in PCa (McVety et al., 2005). The *MLH*1 mutation previously identified in PCa patients comprises the following variants associated with a higher risk of PCa; c.350C>T, c.588+5G>A, c.1537 1547delInsC, c.1667+2delTAAATCAinsATTT, c.1667+2delTAAATCAinsATTT, and c.1732-2A>T (Raymond et al., 2013; Dominguez-Valentin et al., 2016).

#### 4.1.2 MutS homolog 2 (*MSH*2) gene

*The MSH*2 gene is responsible for the production of proteins involved in the MMR mechanism. *MSH2* gene is located in the short arm of chromosome 2 (2p21) and encodes proteins that aid in DNA mismatch repair (Salo-Mullen et al., 2018; Zhen et al., 2018). In collaboration with the MutL homolog (*MLH*1) protein, *MSH2* detects and corrects mismatched DNA base pairs that occur during DNA replication (Chakraborty and Alani, 2016; Furman et al., 2021). The *MSH2* gene is commonly changed in PCa by deletion, missense, and frameshift mutations that also cause the buildup of neoantigens, raise the burden of tumor mutations, and increase the density of lymphocytes that infiltrate tumors (Haraldsdottir et al., 2014; Dominguez-Valentin et al., 2016).

#### 4.1.3 MutS homolog 6 (*MSH6*) gene

The *MSH*6 gene is found on the short arm of chromosome 2 (2p16.3) (Salo-Mullen et al., 2018; Zhen et al., 2018). The *MSH6* gene participates in MMR pathways and mutation of this gene has been associated with an increase in the likelihood of developing PCa (Pritchard et al., 2014) According to recent research, structural rearrangements such as insertions and deletions are what make *MSH*6 more susceptible to mutations (Dominguez-Valentin et al., 2016).

#### 4.1.4 *PMS1* homolog 2 (*PMS2*) gene

The *PMS*2 gene with approximately 38,000 base pairs is located on chromosome 7. The gene consists of 15 exons that code for the 862 amino acids that make up the *PMS*2 protein (Fukuhara et al., 2015). The produced protein is essential to the mismatch repair mechanism, which corrects small insertions and deletions as well as DNA mismatches that may occur during homologous recombination and DNA replication. As previously reported, Genomic integrity is protected by DNA mismatch repair, which corrects mismatches brought on by DNA replication and recombination (Wu et al., 2003). A multitude of essential phases in mismatch repair are orchestrated by the human MutL-alpha heterodimer. As has been previously noted, the nuclear import of MutL-alpha may be the initial regulatory step in initiating the mismatch repair mechanism (Leong et al., 2009). When this protein forms heterodimers with the MutL homolog 1 (*MLH*1) gene product, the resulting structure is known as the MutL-alpha heterodimer. The MutL-alpha heterodimer’s endonucleolytic activity is essential for the removal of the mismatched DNA, and the MutS-alpha and MutS-beta heterodimers identify mismatches and insertion/deletion loops (Reyes et al., 2015). Lynch Syndrome (LS), a multi-organ cancer syndrome, is caused by genetic abnormalities in the four MMR genes (*MLH*1, *MSH*2, *MSH*6, and *PMS*2). The most recent MMR gene to be connected to Lynch Syndrome is *PMS*2 (Hendriks et al., 2006). It has been hypothesized that *PMS2* mutations, in contrast to *MLH1* and *MSH2* mutations, may be linked to a later stage of cancer development (Hendriks et al., 2006). In addition to the several MMR genes, the *PMS*2 gene like many other genes associated with PCa, has also been identified as having polymorphisms and mutations (Fukuhara et al., 2015). Earlier studies have shown that males with *PMS2* mutations are more likely to develop PCa (Haraldsdottir et al., 2014).

### 4.2 Mutation in homologous recombination repair genes

Genomic instability and eventually cancer are brought on by the accumulation of mutations brought on by faults in the DNA damage response pathways (Bartek et al., 2001; Bartek and Lukas, 2003; Antoni et al., 2007), and the path that is frequently blocked is the homologous recombination pathway (van Wilpe et al., 2021). Since some of the proteins in this system are frequently altered in human cancers and a number of heritable conditions that are predisposed to developing cancer, disruption of this route has been demonstrated to play a significant part in the genesis of cancer (Khanna and Jackson, 2001). In the face of various DNA-damaging events, the homologous recombination mechanism is essential for maintaining genomic integrity, Men who carry inherited mutations in essential homologous recombination pathways have a significantly elevated lifetime risk of developing PCa compared to noncarriers (van Wilpe et al., 2021). The ability to test for carriers of those mutations using DNA next-generation sequencing technology has enabled the creation of prospective risk-reduction strategies and the justification for the therapeutic strategy for these individuals. There are currently promising new prospects for treating PCa in people who have mutations in homologous recombination repair genes. The use of immune-based therapies, chemotherapy with platinum, and PARP inhibitors are all viable treatment alternatives (Antonarakis et al., 2019). Castrate-resistant PCa (CRPC) patients have not responded well to immuno checkpoint blocking (ICB) or inhibitor therapy. Only 3%–5% of CRPC patients benefit from anti-PD-1 therapy (Antonarakis et al., 2019). Additionally, research has shown that using ICBs in conjunction with other treatments can improve response rates. For instance, the response rate of a combination of ipilimumab and nivolumab is predicted to be between 10%–26% (Boudadi et al., 2018). When homologous recombination repair genes *BRCA*1/2, *ATM*, *PALB*2, and *CHEK*2 are inactive, a variety of error-prone and non-conservative DNA repair pathways are used, increasing the incidence of cancer and worsening its prognosis while also causing genomic instability (Castro and Eeles, 2012; Amsi et al., 2020). The details of homologous recombination genes involved in DNA damage repair are summarized in Table 2 below.

**TABLE 2:** Summary details of homologous recombination genes related to PCa.

Gene	Chromosome	Exon coverage	Protein	prevalence of Gene mutation in Primary PCa	Prevalence of Gene mutation in m CRPC	References
*BRCA*1	17	24	Breast cancer typer 1 susceptibility protein 1	2.5–6.5	11–13	Dong (2006); Castro and Eeles (2012); Lukashchuk et al. (2023)
*BRCA*2	13	27	Breast cancer typer 1 susceptibility protein 2	2.5–6.5	11–13	Castro and Eeles (2012); Vietri et al. (2021); Xie et al. (2022); Lukashchuk et al. (2023)
*ATM*	11	66	Serine/threonine kinase	0.5–3	4–6	Vietri et al. (2021); Lukashchuk et al. (2023)
*PALB*2	16	13	BRCA2-interacting protein	0–1	0.3–3	Brandão et al. (2020), Lukashchuk et al. (2023)
*CHEK*2	22	14	Serine/threonine kinase	0–1	1.4–2	Lukashchuk et al. (2023); Wang et al. (2015)

#### 4.2.1 *BRCA*1 and *BRCA*2

Epidemiological research has connected Breast cancer gene 1(*BRCA*1) and Breast Cancer gene2 (*BRCA*2) mutations to the risk of PCa; nevertheless, 5%–15% of cases of prostate cancer are caused by high-risk hereditary variables (Ferrís-i-Tortajada et al., 2011). Pathogenic mutations, which account for less than 1% of *BRCA*1, and about 2% in *BRCA2* of incident PCa cases, are likely to be the cause of the disease (Kote-Jarai et al., 2011; Leongamornlert et al., 2012) Click or tap here to enter text in mutation carriers. Male *BRCA*2 carriers have a higher lifetime chance of acquiring PCa than *BRCA*1 carriers (Roy et al., 2012). The DNA damage repair (DDR) process uses non-conservative and potentially mutagenic methods when these *BRCA1* and/or *BRCA2* genes are absent. There is evidence that this genomic instability contributes to the cancer risk associated with harmful *BRCA* gene mutations (Salmi et al., 2021). *BRCA1* and *BRCA2* pathogenic mutation carriers have a relative risk of PCa that is enhanced by 1.8–3.8 and 2.5 to 8.6 times, respectively, by the time they are 65 years old (Thompson et al., 2003; Agalliu et al., 2007; Leongamornlert et al., 2012). *BRCA1* and *BRCA2* gene mutation testing, as well as their correlation with higher Gleason scores (8>), and therapeutic use in PCa management, have drawn the attention of experts to familial PCa in particular (Amsi et al., 2020). Numerous authors have discussed the mutation spectrum in relation to PCa risk, but Ashkenazi Jewish and Icelandic populations have been found to have frameshift deletion 185delAG and a frameshift insertion 5382insC for *BRCA1* and *BRCA2* frameshift deletion, as well as 999del5 founder mutations linked to poor survival in young people (Wilkens et al., 1999; Tryggvadóttir et al., 2007). In addition, PCa patients in the United Kingdom with significantly younger ages (<56 years) were found to have two base pair deletions 5531delTT and four base pair deletions 6710del ACAA frameshift mutation in the *BRCA2* gene (Gayther et al., 2000). Additionally, different studies reported various types and different mutation frequencies (Wilkens et al., 1999; Vazina et al., 2000; Ikonen et al., 2003; Gallagher et al., 2010; Castro et al., 2013; Maia et al., 2016; Shenoy et al., 2016). Most of the reported mutations are frame-shift mutations. The differences in the prevalence of *BRCA1/2* mutations amongst populations may be due to differences in study sample size, inclusion criteria, patient ethnic origins, and other factors. In this review, some of the previously reported *BRCA1* and *BRCA2* pathogenic mutations associated with PCa in different populations are summarized in Table 3.

**TABLE 3:** Mutation spectrum of homologous recombination genes related to PCa.

Gene	Population	Reported Mutation	References
*BRCA1*	Morrocan	c.1953_1956delGAAA	Salmi et al. (2021)
	United Kingdom	c.68_69delAG	Leongamornlert et al. (2012)
		c.212+1G>T	
		c.1954dupA	
		c.2475delC	
	Ashkenazi	*185delAG*	Nastiuk et al. (1999), Petrovics et al. (2019), Wilkens et al. (1999)
		*5382insC*	
	Poland	c.5382insC	Cybulski et al. (2013)
		c.4153delA	
		c. 181T>G	
*BRCA2*	Morrocan	c.7234_7235insG	Salmi et al. (2021)
		BRCA2ΔE12 or BRCA2 del 12	
	*Ashkenazi*	*c.6174delT*	Petrovics et al. (2019), Wilkens et al. (1999)
	United Kingdom	2558insA, c.IVS17-1G>C	(Edwards et al. (2003))
		c.6710delACAA	
		c.7084delAAAAG	
		c.7772insA	
		c.8525delC	
	Icelandic	c.999del5	Tryggvadóttir et al. (2007)
	Turkish	c.4691A>T	Manguoğlu et al. (2010)
	German	c.1813_14_insA	Maier et al. (2014)
		c.3847delGT	
		c.4449delA	
		c.6037A>T	
		c.7495C>T	
*ATM*	Poland	c.1100delC,	Cybulski et al. (2013)
		c.IVS2 + 1G>A	
		c.del5395	
		c.I157T	
		c.8545C>T	Wokołorczyk et al. (2020)
		c.742C>T	
		c.5932G>T	
		c.7096G>T	
		c.8695dupA	
		c.8147 T>C	
		c.7630-2A>C	
		c.7010_7011delGT	
		c.6095G>A	
	China	c.185+1G>C	Ye et al. (2020)
*PALB*2	Poland	c.509_ 510delGA	Wokołorczyk et al. (2020)
		c.172_ 175delTTGT	
		c.509_ 510delGA	
		c.172_ 175delTTGT	
*CHEK*2	USA	c.1100delC	(Dong et al. (2003))
		c.IVS2 + 1G>A	
		c.I157T	
		A751T	
		G715A	
		T470C	
	African American Men	c.1343T>G	Southey et al. (2016)
	European Men	c.1312G>T	Southey et al. (2016)

#### 4.2.2 *Ataxia* telangiectasia mutated (*ATM*) gene

The Ataxia Telangiectasia Mutated (*ATM*) gene is in charge of producing the serine/threonine kinase that is connected to PI3K and is thought to be involved in maintaining genomic integrity. On chromosomes 11q22–23, ATM is located and has 66 exons and a coding sequence of 9,168 base pairs (Choi et al., 2016). In the double-strand break (DSB) repair process, *ATM* functions as a signal transducer and is essential for the detection of DNA damage and the cellular response to it (Virtanen et al., 2019). The rearrangement of antibody genes during B-cell maturation or meiotic recombination, as well as ionizing radiation, chemotherapeutic drugs, or oxidative stress, can all result in DSB, according to other findings (Lieber, 2011; Bednarski and Sleckman, 2012; Choi et al., 2016). RAG DSBs trigger traditional DNA damage responses by activating *ATM* and DNA-PKcs, both of which are serine-threonine kinases that are a member of the phosphatidylinositol-3-kinase (PI3K)-like family (Callén et al., 2009). This is similar to other DSBs created in the G1 phase of the cell cycle (Bednarski and Sleckman, 2012). Both *ATM* and DNA-PKcs function as transducers in the DNA damage response, phosphorylating a range of downstream effectors (Callén et al., 2009).

Due to the different and redundant functions that *ATM* and DNA-PKcs play in the response to RAG-mediated DSBs, immune system deficiencies and errors in DNA end repair occur in mice and individuals who lack either of these kinases (Bednarski and Sleckman, 2012). According to the information that is currently available, castration-resistant PCa have an enrichment of approximately two times the frequency of localized PCa in terms of tumoral germline or somatic *ATM* mutations, which vary from 5% to 8% of the tumors, also Men with pathogenic *ATM* mutations have an increased risk of developing PCa, which may potentially cause the condition to appear earlier (Giri and Beebe-Dimmer, 2016; Thalgott et al., 2018; Wokołorczyk et al., 2020). There is limited information about populations in Africa, despite some populations being known to carry these mutations and being discussed in this review. In the research conducted on 390 Polish people, eight genes were mutated and 76 males (19.5%) had a mutation in one of the *BRCA1*, *BRCA2*, *ATM*, *CHEK2*, *MSH2*, or *MSH6* genes. A total of 11 mutations (2.8%) associated with *ATM* were among the reported gene variations. A few one-stop gains in the *ATM* gene have been documented, including c.8545C>T, c.742C>T, c.5932G>T, and c.7096G>T. A frameshift mutation, c.7010_7011del, two splice acceptor variations, c.7630-2A>C, and two missense mutations, c.6095G>A and c.8147 T>C, are all found in the *ATM* gene, but all of those alterations were not reported in the control groups (Wokołorczyk et al., 2020).

#### 4.2.3 Partner and localizer of *BRCA2 (PALB2)*

The BRCA1-PALB2-BRCA2 protein, which is encoded by the partner and localizer of *BRCA2* (PALB2), is found on chromosome 16 and forms the *PALB2* complex. The molecular scaffold, *PALB2*, interacts with *BRCA2*. It is essential for homologous recombination and DNA double-strand break (DSB) repair (Sy et al., 2009). Fanconi anemia is also brought on by germline homozygous loss of function (LoF) mutations of *PALB2,* like *BRCA2* (Reid et al., 2007; Xia et al., 2007)*,* although heterozygous LoF mutations have been associated with hereditary breast cancer and pancreatic cancer (Jones et al., 2009; Antoniou et al., 2014). Despite new correlations between pathogenic *PALB2* mutations and an elevated risk of different cancer, research on the *PALB2* gene’s role in PCa has produced conflicting results. The authors of this study are aware of new results linking *PALB2* to a statistically significant increase in the chance of developing PCa (Yang et al., 2020). While it has long been known that these polymorphisms increase the risk of breast cancer, data on *PALB2*’s role in PCa have also been decisively proven (Horak et al., 2019; Wokołorczyk et al., 2020; Bouras et al., 2022).

The authors also acknowledge that there has not been much research on *PALB2* pathogenic mutations in PCa patients, but research conducted in Poland in 2021 by Wokolorczyk and others found that aggressive cancers with high Gleason scores of 8–10 were more frequently diagnosed with the two founder mutations of *PALB2* c.509_510delGA and c.172_ 175delTTGT, which together account for 80% of all *PALB2* mutations. Furthermore, the 5′ ends of the *PALB2* gene were the location of these founder mutations, as described by Wokolorczyk (Wokołorczyk et al., 2021). Additionally, these c.509_510delGA and c.172_ 175delTTGT variants result in a translational frameshift with a predicted alternate stop codon and are predicted to cause *PALB2* function to be lost through premature protein truncation or nonsense-mediated messenger RNA decay. These research findings are strengthening the case that the two Polish founder mutations of *PALB2* are also pathogenic for breast cancer (Wokołorczyk et al., 2021).

#### 4.2.4 Checkpoint kinase 2(*CHEK2)*

Cell cycle checkpoint kinase 2 (*CHEK*2) is a protein that participates in DNA damage response in many distinct cell types. Ataxia Telangiectasia Mutated (*ATM*) protein activates *CHEK*2 to prevent the buildup of mutations and reduce the risk of cancer when DNA is damaged (Bartek et al., 2001; Bartek and Lukas, 2003; Antoni et al., 2007). By activating *CHEK*2 in response to DNA damage, the checkpoint control pathways’ downstream targets, such as p53, Cdc25A, Cdc25C, *BRCA1*, E2F1, Pml1, Plk3, and other substrates, are signaled. These substrates altered biological activities resulting in cell cycle blockage, enhanced DNA repair, or apoptosis (Bartek et al., 2001; Bartek and Lukas, 2003; Antoni et al., 2007). Cell cycle regulator *CHEK*2 controls the homologous recombinant DNA repair process, suppresses tumors, and genetic changes in it render cancers more susceptible to more advanced targeted therapies. *CHEK*2 is phosphorylated and activated in an *ATM-*dependent way in response to certain DNA-damaging substances (Matsuoka et al., 1998).

In the cell-cycle checkpoint control, active *CHEK*2 and other DNA damage-triggered protein kinases stabilize TP53 or speed up Cdc25A degradation through the coordination of DNA repair, cell-cycle progression, and death (Matsuoka et al., 1998; Hirao et al., 2000; Falck et al., 2001; Zannini et al., 2014) The tumor-suppressing function of the cell cycle regulator *CHEK*2 is mediated via homologous recombinant DNA repair, and genetic changes in it render malignancies susceptible to more advanced targeted therapies. *CHEK*2 is phosphorylated and activated in an *ATM*-dependent way in response to certain DNA-damaging agents (Matsuoka et al., 1998). Activated *CHEK*2 and other DNA damage protein kinases stabilize TP53 or hasten Cdc25A degradation in the cell-cycle checkpoint regulation via coordinating DNA repair, cell-cycle progression, and apoptosis (Matsuoka et al., 1998; Hirao et al., 2000; Bartek et al., 2001; Falck et al., 2001; Zannini et al., 2014) The frameshift 1100delC mutation in the *CHEK2* gene, which causes the translation of a truncated *CHEK2* protein product lacking kinase function, is the gene variation that has received the most attention. A substitution G>A in the exon 2 splice site has also been known as the IVS2 + 1G>A (or 444 + 1G>A) mutation. Because of this substitution, an aberrant splicing event occurs which also causes the insertion of a 4-base pair fragment, and finally, a frameshift that terminates at codon 154 in exon 3 (Dong et al. (2003)). Men with the *CHEK2* 1100delC, IVS2 + 1G>A, and I157T missense mutations have a higher chance of developing PCa, while there is no evidence that these *CHEK2* 1100delC, variants cause the disease in all cases of familial PCa. A previous study using 178 patients identified 13 *CHECK2* mutations, of which 9 (10.7%) were found in the prostate cancer of 84 unselected patients and 4 (4.3%) were found in the early-onset cancer of 94 patients (Dong et al. (2003)). Men from both Europe and Africa have shown similar evidence of a *CHEK2* risk mutation for PCa. African men had a *CHEK2* c.1343T>G OR of 3.03 (95% CI 1.53 to 6.03, *p* = 0.0006), whereas European men had a *CHEK2* c.1312G>T OR of 2.21 (95% CI 1.06 to 4.63, *p* = 0.030) (Southey et al., 2016).

## 5 Genetic mutation and tumor antigenicity in prostate cancer

The tumor is more likely to be recognized by the immune system when it contains more neoantigens. The accumulation of these neo-antigens is triggered by mutations in the DNA repair process. The prevalence of mutations, which might increase the number of neoantigens, would increase if specific DNA repair mechanisms, including MMR and HRR are altered (Jiricny, 2013; De Mattos-Arruda et al., 2020; Golan et al., 2021; Ma et al., 2022; Amodio et al., 2023). Neoantigen-reactive T cells have been demonstrated in studies to be one of the critical components of immunotherapy efficacy, particularly in tumors with a high tumor mutational burden (TMB) (Ye et al., 2020). When ICBs like CTLA4 and PD1 are used to treat some forms of cancer, tumors with high TMB have superior clinical results (Lu and Robbins, 2016; Ye et al., 2020; Graf et al., 2022; Shi et al., 2022) Cancer-associated antigens, including neoantigens derived from genetic mutations, are presented to CD8^+^ T cells through the major histocompatibility complex (MHC) on dendritic cells (DCs), and professional antigen-presenting cells (APCs). However, the majority of neoantigens are usually not recognized by the immune system, thus identification of highly tumor-specific antigens is important for the development of personalized immunotherapy (Kiyotani et al., 2018; De Mattos-Arruda et al., 2020) In patients with advanced and other types of cancers, greater neoantigen load is a predictive factor for a better result when utilizing ICBs (Hopfner and Hornung, 2020). Further studies are needed to fill this knowledge gap because there is a lack of enough evidence in the literature to relate specific DDR gene mutations to TMB, resistance to DNA-damaging treatments, or radiation therapy.

## 6 Clinical and therapeutic implications of DNA damage response mutation in prostate cancer

In order to detect PCa and increase survival rates without over diagnosing and overtreating patients, screening techniques have been developed (Force, 2018). Oncologists can use risk stratification in therapeutic decision-making, and the stratification groups are routinely used to establish the inclusion or exclusion criteria for patients to take part in studies looking at drugs that are specifically targeted for a given risk group (Rodrigues et al., 2012). There are a number of prostate risk stratification methods in place, including the Gleason score, PSA levels, clinical staging, and histological staging, among others; however, none of these are enough to predict outcomes (S.-Y. Wang et al., 2014). As a result, this drove the need for additional research until recently, when 333 primary prostate tumors were examined for the first time by the Cancer Genome Atlas Research Network, which discovered that 19% of them contained DNA repair gene abnormalities (“Cancer Genome Atlas Research Network,” 2015). New classification schemes based on molecular traits have been described, which will improve PCa precision medicine. Recent studies have demonstrated that the DDR gene mutation raises the risk of PCa (Castro et al., 2019; Chung et al., 2020).

Pathogenic mutation in the DDR genes *BRCA1/2* and *ATM* status differs between risk for aggressive and indolent PCa and is linked to a younger death age and a shorter survival period (Na et al., 2017). According to research, while some people have aggressive cancers that metastasize and result in disease-related death, many with indolent tumors can be successfully treated with early therapy or monitored (Sakellakis et al., 2022). Furthermore, studies conducted in various contexts with various populations have demonstrated that homologous recombination repair (HRR) and mismatch repair genes, which are mutated in a high proportion of patients, are involved in aggressive PCa biology (Lukashchuk et al., 2023). When managing familial cancer risk, knowledge of the therapeutic sensitivity to novel targeted medications imparted by these mutations in DNA damage response could be life-saving (Herberts et al., 2023; Lukashchuk et al., 2023; Sorrentino and Di Carlo, 2023).

### 6.1 Genetic mutation as prognostic biomarkers

Patients with homologous recombination repair (HRR) and mismatch repair (MMR) gene mutations are more likely to have poor clinical outcomes, intraductal and cribriform structure, and higher Gleason scores than patients without these mutations (Risbridger et al., 2015; Schweizer et al., 2019). According to studies, localized PCa with mutations in the HRR and MMR genes displays a biological profile that is very aggressive and resembles a treatment-resistant metastatic disease. The dysregulation of MED12L/MED12 in localized PCa with HRR and MMR gene alterations is an illustration of this. A combination of genomic dysregulation of the Wnt-pathway mediator complexes and enhanced genomic instability can account for this. This imbalance is a typical castration-resistant PCa molecular feature (Taylor et al., 2017). Genomic instability, which is a characteristic of cancer cells that is not present in normal cells due to DDR defects, is a well-known characteristic of these cells. Men with genomic instability seem to have a worse prognosis and are more likely to be diagnosed with PCa, especially in somatic cells with shorter telomere lengths (Heaphy et al., 2013). According to the most recent study that thoroughly investigated the function of DDR mutation in PCa survival (Zhang et al., 2022), the majority of DDR pathway mutations have been reported to be linked to a poor prognosis (Heaphy et al., 2013). Castro and others highlighted the function of the DDR gene mutation as a predictive biomarker after finding a substantial relationship between germline *BRCA1/2* gene mutation and PCa aggressiveness. In hereditary *BRCA1/2* mutation carriers, Castro found that the risk of stage T3/T4 cancer, nodal involvement, a Gleason score of 8 or higher, and metastases at the time of initial diagnosis was increased and that the survival time was also decreased (Castro et al., 2013). In another study, it was discovered that patients with lethal PCa had a significantly higher combined rate of germline *BRCA/ATM* alterations than those with localized PCa, and those with local disease or a diagnosis of metastases had a shorter prostate-cancer-specific survival time than non-carriers (Na et al., 2017). These findings were recently supported by research conducted by Lukashchuk, Zhang, Silvestri, and others (Silvestri et al., 2020; Zhang et al., 2022; Lukashchuk et al., 2023) thus, in addition to other reported findings elsewhere, the current data suggest that DDR gene mutation would be a helpful marker for disease screening. Additionally, among reported DDR genes, the *CHEK2* mutation provides reliable evidence of PCa risk in African men (Southey et al., 2016).

### 6.2 Genetic mutation and prostate tumor recognition of the adaptive immune response

The antigen recognition and immune cell activation in the adaptive immune system depends on the interaction between molecules of MHC on the surface of cells that present antigen (APCs) and costimulatory molecules found on the surface of naive T cells (Jiang et al., 2021). According to previous research, antigen recognition by MHC molecules of Intratumoral T cell lymphocytes depends on the activation and amount of T cells (Alexandrov et al., 2013; Jiang et al., 2021). As reported by Alexandrov and others (Alexandrov et al., 2013), the number of mutations that cause alterations in the sequence of amino acids of the protein in a tumor has a positive correlation with the quantity of tumor neoantigen. Prostate and various forms of cancer develop at various rates in terms of intra-tumoral T-cell activation and the number of cells involved (Alexandrov et al., 2013). Additionally, when the non-synonymous mutation occurs in DDR genes, the newly produced peptide is identified as foreign by immune cells and elicits an adaptive immune response (Riaz et al., 2016). These non-synonymous mutation rates could rise as a result of DDR deficits and they have also been linked to the synthesis of peptides that affect the proinflammatory responses, particularly in MMR genes (Jiricny, 2013).

### 6.3 Genetic mutation in predicting immunotherapy

Immune Checkpoint Blockades (ICB)are therapeutic drugs that specifically target negative inhibition receptors on host T lymphocytes, such as cytotoxic T lymphocyte antigen 4 (CTLA-4) and programmed death 1 (PD1), which are frequently taken over tumors to hinder an efficient antitumor immune response. ICB acts on the immune system to strengthen antitumoral immunity, in contrast to conventional cancer therapies, which directly target cancer cells (Lei et al., 2021). The therapeutic efficacy of ICB using antibodies against inhibitory compounds produced on tumor and immune cells has been shown for a wide range of tumor types. Recent studies have demonstrated the efficacy and approval of anti-CTLA-4, anti-PD1, anti-PDL1, and their combinations with other medications. Compared to chemotherapy alone, the administration of these ICB has considerably reduced the incidence of cancer (Wei et al., 2018; Akinleye and Rasool, 2019). The findings indicate that not all PCa patients who are not actively selected benefit from ICB because of the diversity of the disease and therapy response. Unfortunately, the exact reason why different people respond to ICB therapy in different ways is still unknown. Additionally, while employing ICB, increased cancer treatment costs and unnecessary immune-related side effects should be considered (Lei et al., 2021). However, significant research is needed to address inherent and acquired problems that limit the number of individuals who benefit from ICB. Similarly, for efficient utilization of ICBs, it will be necessary to identify potential candidates for therapies. This helps to determine whether a patient will be a long-term responder, a short-term responder, or a non-responder (Lei et al., 2021). Through the use of these biomarkers, we would be able to treat responders to the fullest extent possible without unnecessarily harming non-responders (Lei et al., 2021).

Checkpoint inhibitors have also been demonstrated to be beneficial for patients with advanced PCa (Teply and Antonarakis, 2017; Boudadi et al., 2018; Hansen et al., 2018; Antonarakis et al., 2020). In men with *BRCA1*/*2* mutations, subset analysis of larger trials revealed a greater effect for single-agent PD-1 inhibitors. For instance, in a clinical trial on pembrolizumab monotherapy for PCa, Antonarakis, and others found that men with *BRCA1/2* or *ATM* mutations had an objective response rate of 12%, which was much greater compared to the 4% objective response rate seen in men with no those mutations (Antonarakis et al., 2020). Three of the five men (60%) who had tumors with altered HRR genes (*BRCA1/2*) demonstrated objective responses in previous clinical trials in 78 men with mCRPC treated with the combination of ipilimumab and nivolumab (Sharma et al., 2020). Similar results were also reported from a study carried out by Boudadi at John Hopkins (Boudadi et al., 2018).

### 6.4 Mutation in DNA damage as biomarkers for response to poly (ADP-ribose) polymerase inhibitors

The amount of DNA damage brought on by internal and external sources would be fatal in the absence of the DNA damage repair (DDR). In order to prevent damage from being duplicated during the S-phase or transferred to the daughter cells during mitosis, the DDR evolved as a structured network of pathways that repair DNA and stop cell division. Hanahan and Weinberg assert that DDR gene alterations can cause genomic instability and mutations that may result in cancer (Hanahan and Weinberg, 2011). Additionally, active oncogenes enhance replication stress by driving cells into the S-phase before they are ready. This leads to DNA sequence alterations and aberrant DNA structures that need to be resolved by the DDR (Saxena and Zou, 2022). According to Curtin, a mutation in one DDR gene that is associated with the DDR pathway may result in enhanced activity of compensatory pathways, leading to resistance to DNA-damaging radiation therapy and chemotherapy (Curtin, 2023). In order to treat PCa, the development of drugs that target the DDR was initially justified by the need to get around these mechanisms of resistance. However, the approach used by chemo- or radiotherapies to stop the DDR also causes more harm to healthy cells while maintaining increased antitumor effects (Curtin, 2012). Previous researchers suggested the inhibition of PARP, which interferes with DNA damage repair and results in tumor cell death, is connected to the anticancer mechanism of medicines targeting DDR. While PARP inhibition is tolerated in normal cells, it has a significant impact on tumor cells with HRR mutations (Keung et al., 2019; Lang et al., 2019; Rose et al., 2020).

Single-strand breaks (SSB) which occur as a result of PARP inhibition facilitate the formation of damage in DSB which is mainly repaired by HRR (Yi et al., 2019). The coincident loss of PARP function in cancer cells with changed HRR proteins engaged in HRR inadequate repair leads to the formation of Double strand breaks (DSBs) and consequently cell death (Yi et al., 2019). Authors have recently reported a breakthrough in PCa treatment that uses the DDR gene mutation to target the tumor only while protecting healthy cells with intact DDR pathways. This discovery has led to numerous clinical trials that take advantage of these defects, and numerous PARP inhibitors, such as olaparib, rucaparib, niraparib, talazoparib, and veliparib, have been successfully tested in patients with a variety of DDR gene mutations, including *BRCA1*, *BRCA2*, *ATR*, *ATM*, *CHEK1/2*, and *PALB2* (Catalano et al., 2023).

In recent years, the PCa treatment PARPi—poly (ADP-ribose) polymerase inhibitors—has been approved by the FDA (Catalano et al., 2023). Patients with mutations in the DDR response genes *BRCA2, ATM*, and *BRCA1* reported significantly greater response rates to olaparib than noncarriers in one of the earliest trials of PARPi in patients with advanced PCa (Gurley and Kemp, 2001). Similar to this, rucaparib’s phase II TRITON2 research results by Abida and others hastened its approval after demonstrating a 51% radiographic response rate for docetaxel-resistant patients with BRCA1/2 mutation in mCRPC (Abida et al., 2020). Researchers have recently demonstrated that next-generation hormonal drug combined with PARPi therapy for mCRPC provides the strongest PARPi benefit (Agarwal et al., 2022; Chi et al., 2022; Clarke et al., 2022; Herberts et al., 2023).

## 7 Conclusion

Despite the difficulties and variations in PCa diagnosis and treatment that have been mentioned, the most current study is showing gaps in the recognized therapy guidelines. To better diagnose and treat PCa patients, a multidisciplinary approach involving molecular biologists, oncologists, and immunologists is necessary. Research on effective and targeted treatments for prostate cancer is therefore critically needed, as well as investigations on the variety of genetic abnormalities prevalent in prostate cancer. Future studies should focus on generating genetic data that could be used to improve the health of PCa patients through immunologically based targeted therapies or combination therapeutic options, particularly in African settings where knowledge of the genetic spectrum of genes associated with the disease is limited.

## Abbreviations

ADT, Androgen deprivation therapy; PCa, Prostate cancer; BER, BER base excision repair; *BRCA1*, Breast cancer gene 1; *BRCA2*, Breast cancer gene 2; CRPC, Castration-resistant prostate cancer; DDR, DDR DNA damage repair; HRR, Homologous recombination repair; mCRPC, Metastatic castration-resistant prostate cancer; MMR, Mismatch repair; NCCN, National Comprehensive Cancer Network; PARP, Poly (ADP-ribose) polymerase; PARPi, Poly (ADP-ribose) polymerase inhibitor; PSA, Prostate-specific antigen; DSBs, Double strand breaks; SSBs, SSBs single-strand breaks.
